# Meta-Analysis of Three Different Types of Fatigue Management Interventions for People with Multiple Sclerosis: Exercise, Education, and Medication

**DOI:** 10.1155/2014/798285

**Published:** 2014-05-14

**Authors:** Miho Asano, Marcia L. Finlayson

**Affiliations:** School of Rehabilitation Therapy, Queen's University, Louise D. Acton Building, 31 George Street, Kingston, ON, Canada K7L 3N6

## Abstract

Fatigue is a common symptom of multiple sclerosis (MS) with negative impacts extending from general functioning to quality of life. Both the cause and consequences of MS fatigue are considered multidimensional and necessitate multidisciplinary treatment for successful symptom management. Clinical practice guidelines suggest medication and rehabilitation for managing fatigue. This review summarized available research literature about three types of fatigue management interventions (exercise, education, and medication) to provide comprehensive perspective on treatment options and facilitate a comparison of their effectiveness. We researched PubMed, Embase, and CINAHL (August 2013). Search terms included multiple sclerosis, fatigue, energy conservation, Amantadine, Modafinil, and randomized controlled trial. The search identified 230 citations. After the full-text review, 18 rehabilitation and 7 pharmacological trials targeting fatigue were selected. Rehabilitation interventions appeared to have stronger and more significant effects on reducing the impact or severity of patient-reported fatigue compared to medication. Pharmacological agents, including fatigue medication, are important but often do not enable people with MS to cope with their existing disabilities. MS fatigue affects various components of one's health and wellbeing. People with MS experiencing fatigue and their healthcare providers should consider a full spectrum of effective fatigue management interventions, from exercise to educational strategies in conjunction with medication.

## 1. Introduction

Fatigue is one of the most common and devastating symptoms of multiple sclerosis (MS) with negative impacts extending from general functioning to quality of life [1–4]. Both the cause and consequences of MS fatigue are considered multidimensional [5, 6] and necessitate multidisciplinary treatment for successful symptom management [6]. Clinical practice guidelines suggest medication (e.g., Amantadine and Modafinil) and rehabilitation (e.g., exercise, energy or fatigue self-management education, and cognitive behavioral therapy) for managing fatigue [6, 7].

Several published reviews have examined the effectiveness of individual types of fatigue management interventions for people with MS. For example, reviews of pharmacological intervention trials (i.e., Amantadine and Modafinil) on fatigue in MS noted that the current evidence is weak and inconclusive [8, 9]. In comparison, recent reviews of exercise training [10], energy management education [11], and cognitive behavioral therapy (CBT) [12] suggest that these interventions may be beneficial for MS fatigue management.

The body of research investigating the effect of MS fatigue management interventions is growing, but to our knowledge, no review to date has examined the individual types of interventions together for the purpose of comparison. Such a review would provide a comprehensive perspective on treatment options and facilitate a comparison of their effectiveness. Therefore, this review pursued four questions among adults with MS. (1) How effective are exercise interventions for reducing the impact or severity of self-reported fatigue? (2) How effective are educational programs for reducing the impact or severity of self-reported fatigue? (3) How effective are commonly used fatigue medications (Pharmacological intervention trials) for reducing the impact or severity of self-reported fatigue? And (4) are MS fatigue rehabilitation interventions (i.e., exercise and educational programs) more effective than the common MS fatigue medications?

## 2. Methods

### 2.1. Data Sources and Search Strategies

We researched PubMed, Embase (Ovid), and Cumulative Index to Nursing and Allied Health Literature (CINAHL, EBSCO) up to August 2013 for original articles written in English and published in a peer-reviewed journal. Gray literature (e.g., abstracts, conference proceedings, and editorials) and existing reviews (i.e., narrative, scoping, and systematic reviews) were excluded from the review. Search terms included multiple sclerosis, fatigue, energy conservation, Amantadine, Modafinil, and randomized controlled trial. Subject heading, keyword, and publication type searches were performed, each of which varied slightly depending on the term mapping of the database.

### 2.2. Criteria for Considering Trials for This Review

#### 2.2.1. Study Design

The review was limited to randomized controlled trials (RCTs). Trials had to include at least two data points (i.e., pre- and postintervention assessments) for the mean and standard deviation (SD), standard error (SE), or 95% confidence interval (CI) of the outcome. Trials that focused only on investigating the moderating or mediating factors related to the outcome or the long-term effect of an intervention were excluded from the review.

#### 2.2.2. Study Participants

No restrictions were set for type of MS, disease severity, level of disability, or sex of study participants. Studies were excluded if they targeted nonadults (age younger than 18 years old) or included populations other than MS.

#### 2.2.3. Interventions

Different types of rehabilitation and pharmacological interventions can be prescribed for MS fatigue management. Trials had to include a purpose statement clearly indicating that the intervention targeted fatigue/energy in MS. For rehabilitation interventions, only interventions commonly available in traditional rehabilitation settings and offered by rehabilitation professionals (e.g., occupational or physical therapists, nurses, psychologists, and physiatrists) were included in this review (e.g., exercise or physical therapy, educational, self-management program, and psychotherapy). Trials examining the effect of supplements or interventions that would be atypical in traditional rehabilitation settings were excluded from the review (e.g., bee sting or venom therapy and transmagnetic stimulation field). Amantadine and Modafinil are considered two of the most common fatigue medications prescribed in MS [9, 13]. For pharmacological interventions, only Amantadine and Modafinil were included in this review.

#### 2.2.4. Outcome Measures

Only trials that administered a patient-reported outcome measure of the impact or severity of fatigue were included in the review. When trials administered multiple measures related to fatigue, a measure that was developed for, validated, and commonly used in MS (i.e., Fatigue Severity Scale (FSS) [14], Modified Fatigue Impact Scale (MFIS) [15], and Fatigue Impact Scale (FIS) [1]) was selected for the analysis, unless noted otherwise.

Two reviewers independently evaluated the retrieved citations for relevance by examining the title and abstract based on the abovementioned review criteria (i.e., study designs, study participants, interventions, and outcomes) and created a selection list to compare. Any disagreements on relevance were resolved by discussions.

### 2.3. Risk of Bias Assessment

The Cochrane's risk of bias tool was used to assess the quality of the included trials [16]. The tool assesses selection bias, performance bias, detection bias, attrition bias, and reporting bias. Having used the tool, we must acknowledge that blinding participants to the study hypothesis, design, and intervention in rehabilitation research is extremely difficult (i.e., high risk) since active involvement of the participant is required (e.g., must attend a session, engage in activities with therapists, etc.) [17]. Concurrently, blinding of outcome assessment is also considered extremely difficult when patient-reported outcome measures are used (i.e., high risk). Furthermore, despite many trials being described as single- or double-blind studies, the blinding was often unspecified in methods. Although two reviewers completed the risk of bias assessment independently, compared the results, and resolved any disagreements by discussions, the results were not used to critique the included trials or to finalize the selection (Table 4).

### 2.4. Data Analysis

Once the selection process was finalized, trials were first grouped into one of three major intervention foci: (i) exercise (e.g., physical and exercise therapy); (ii) educational (e.g., cognitive behavioral therapy, patient education, and self-management programs); or (iii) pharmacological (i.e., Amantadine and Modafinil). Within each focus, trials were then grouped by the outcome measure (i.e., Modified Fatigue Impact Scale (MFIS) [15], Fatigue Impact Scale (FIS) [1], Fatigue Severity Scale (FSS) [14], and other) for the presentation.

There are different ways to calculate ES [18]. Because this review included only RCTs, ES was calculated by taking the mean difference in the change (pre- and postintervention) in the outcome measure between the two groups (experimental versus comparison group) divided by the initial pooled standard deviation. When trials had multiple pre- and postintervention assessments, the data from the initial preintervention and the first postintervention assessment were used to calculate ES. If trials used a delayed or waitlisted comparison group or a crossover design, only the data from the initial pre/postintervention period were used to calculate ES, unless noted otherwise. When trials presented insufficient data for ES calculation (e.g., no presentation of means, SDs, SE, and/or 95%CI), the first author of that trial was contacted once via email for the information. When the necessary information was not obtained from the author, the study was excluded from the review.

A positive ES favors an intervention group, and a negative ES favors a comparison group. ES ≥ 0.80 is considered strong; 0.50 ≤ ES ≤ 0.79 is considered moderate; 0.20 ≤ ES ≤ 0.49 is considered weak [19, 20]. In this review, ES was considered statistically significant if its 95% confidence interval (CI) excluded the null value of zero.

### 2.5. Heterogeneity and Publication Bias

The standard *Q* test and* I* ^2^ statistics were used to examine the heterogeneity among trials [21]. A significant *Q* test suggests the presence of heterogeneity and the* I* ^2^ estimates the magnitude of heterogeneity. When the presence of heterogeneity among the data is detected, the random-effects model is recommended for the analysis of pooled intervention effect [22]. As the issue of heterogeneity in the contents of rehabilitation interventions within the same focus has been acknowledged in similar reviews [23, 24], the pooled ES was calculated using the random-effects model. The Begg-Mazumdar rank correlation test and Egger intercept test were used to examine the publication bias. A significant correlation or intercept (*P* < 0.05) suggests the presence of publication bias.

## 3. Results

**Figure 1:**
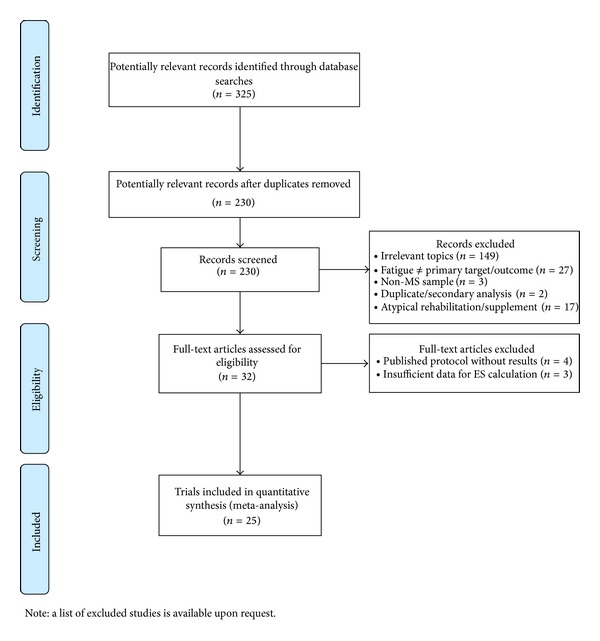
Review flow diagram.

The initial search identified 230 citations. After the full-text review, 25 RCTs met all inclusion criteria and were maintained for the review process [25–49].  Figure 1 presents the process of selecting 25 RCTs included in this review.

### 3.1. Trials and Interventions

Eighteen rehabilitation intervention trials (ten exercise intervention trials [25–34] and eight educational intervention trials [35–42]) and seven pharmacological intervention trials [43–49] targeting fatigue were selected. A wide range of exercise interventions were prescribed (e.g., aerobic, aquatic, and inspiratory muscle exercise; vestibular rehabilitation program; progressive resistance training; climbing; and yoga). Educational interventions included group fatigue/energy management programs and psychotherapies (e.g., cognitive behavioral therapy and mindfulness-based intervention). The majority of pharmacological intervention trials examined the effect of Amantadine (*n* = 5/7, 71%).

### 3.2. Participants

The number of participants who entered trials per intervention group ranged from 7 to 115. For all three types of interventions, the participants were predominately women. The trials reported the mean age of the participants from 25 (a pharmacological trial) to 56 years old (an educational intervention trial). The participants in the pharmacological and exercise intervention trials reported minimal to moderate disability commonly assessed by the Kurtzke Expanded Disability Status Scale (EDSS) [50, 51], whereas the participants in the educational intervention trials reported mild to severe disability. The educational intervention trials included more participants with progressive MS. Collectively the data obtained from 1499 participants from 25 trials were included in the review.

### 3.3. Outcome Measures

The Fatigue Severity Scale (FSS) was the most common outcome measure administered (*n* = 11/25, 44%) in included trials, followed by the Modified Fatigue Impact Scale (MFIS) (*n* = 8/25, 32%) and the Fatigue Impact Scale (FIS) (*n* = 2/25, 8%). The exercise and pharmacological intervention trials favored the FSS whereas the educational intervention trials favored the MFIS.

### 3.4. Effect of Exercise Interventions

ES for the exercise interventions ranged from −0.24 (95%CI: −1.15 to 0.64) to 2.05 (95%CI: 1.00–3.11). After taking 95%CI into consideration, three studies (30%) presented a significant intervention effect [25, 26, 28]. The pooled ES was 0.57 (95%CI: 0.10–1.04, *P* = 0.02) with the pooled sample sizes of 112 for the experimental groups and 121 for the comparison groups [25–34]. The presence of heterogeneity among the exercise intervention trials was detected by the *Q* test (*P* = 0.003) and* I* ^2^ of 65%. The Begg-Mazumdar and the Egger test for publication bias were not significant.

### 3.5. Effect of Educational Interventions

ES for the educational interventions ranged from −0.16 (95%CI: −0.72 to 0.38) to 1.11 (95%CI: 0.43 to 1.78). After taking 95%CI into consideration, six studies (75%) presented a significant intervention effect [35, 36, 38, 40–42]. The pooled ES for the educational interventions was 0.54 (95%CI: 0.30–0.77, *P* < 0.001) with the pooled sample sizes of 338 for the experimental groups and 324 for the comparison groups [35–42]. The presence of heterogeneity among the educational intervention trials was detected by the *Q* test (*P* = 0.04) and* I* ^2^ of 50%. The Begg-Mazumdar and the Egger test for publication bias were not significant.

### 3.6. Effect of Pharmacological Interventions (Amantadine and Modafinil)

ES for the pharmacological interventions ranged from −0.59 (95%CI: −1.26 to 0.06) to 0.55 (95%CI: −0.06 to 1.16). After taking 95%CI into consideration, one study (14%) presented a significant intervention effect [48]. The pooled ES for the pharmacological interventions was 0.07 (95%CI: −0.22–0.37, *P* = 0.63) with the pooled sample sizes of 303 for the experimental groups and 301 for the comparison groups [43–49]. The presence of heterogeneity among the pharmacological intervention trials was detected by the *Q* test (*P* = 0.004) and* I* ^2^ of 67%. The Begg-Mazumdar and the Egger test for publication bias were not significant.

**Figure 2:**
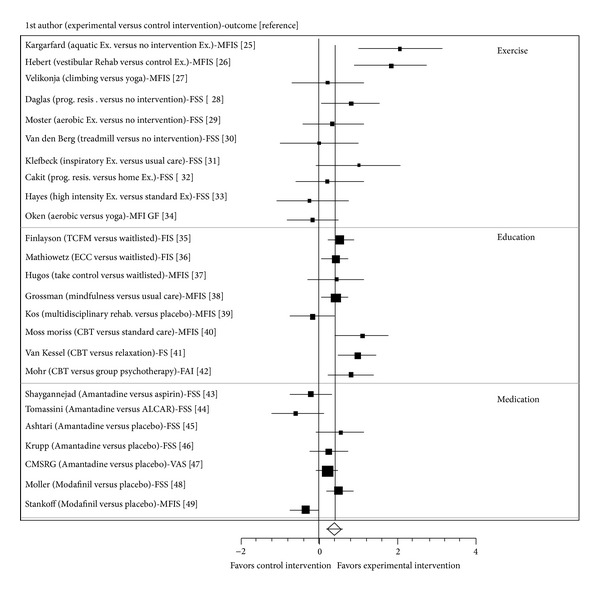
Forest plots.

Table 1 presents a summary of intervention effects.  Figure 2 and Table 2 present the combined forest plots of all trials for a visual comparison and a summary table of ES and 95%CI for each trial.  Table 3 presents a brief summary of active interventions and their participants of trials included in the review.

## 4. Discussion and Conclusion

Based on this review of MS fatigue management interventions, rehabilitation interventions (both exercise and educational interventions) appear to have a stronger and more significant effect on reducing the impact or severity of patient-reported fatigue compared to the two most commonly prescribed fatigue medications (i.e., Amantadine and Modafinil). These results suggest that rehabilitation interventions should be the initial treatment choice for people with MS who are reporting disabling fatigue. This recommendation takes a different perspective than current MS research and care, in which rehabilitation is often considered as an alternative or supplemental treatment option relative to medication.

The evidence from our review points to the potential for exercise interventions to be beneficial for managing MS fatigue, which is consistent with findings from Pilluti et al.'s work [10]. Yet, the extent of the intervention effects varies considerably and only a certain group of people with MS appear to experience benefit. Therefore, the extent to which the effectiveness of exercise interventions extends to other MS subgroups, for example, older adults with MS or those with progressive MS and/or severe disability, is unknown. Examining individual ES, only three out of ten included exercise trials (30%) were significant. All three interventions were different (i.e., aquatic exercise, vestibular rehabilitation, and progressive resistance training). Consequently, it is not possible to identify what types of exercise or what components of exercise should be included in an intervention to achieve positive fatigue management benefits. Future trials with different subgroups and exercise formats are warranted.

Exercise interventions in this review presented the largest pooled ES but with the widest 95%CI, likely due to the small samples and lack of fatigue screening for study eligibility. Only one of ten exercise trials (10%) clearly stated that the participants were screened for the presence of fatigue as a study eligibility criterion, whereas the majority of participants in the educational and the pharmacological intervention trials (75% and 100%, resp.) completed such screening. Furthermore, the current evidence does not explain how the presence or the level of fatigue prior to commencing interventions influences the outcome.

Educational intervention trials presented the pooled ES similar to the exercise intervention trials but had the narrowest 95%CI. Furthermore, six out of eight trials (75%) were significant. These educational intervention trials included samples that tended to be less homogenous relative to the samples in the exercise trials. Therefore, a variety of groups of people with MS (including older adults and those with progressive MS or with severe disability) are likely to experience the benefit.

Pharmacological intervention trials presented the smallest effect, which was nonsignificant pooled ES with a relatively narrow 95%CI. Only one out of seven trials (14%) was significant. These pharmacological intervention trials included a larger number of samples that were homogenous. Agreeing with the existing evidence [8, 9], the effect of pharmacological interventions on MS fatigue found in this review is weak and inconclusive. The search strategy and selection criteria for RCTs that assessed the effect of pharmacological interventions on MS fatigue were restricted to Amantadine and Modafinil. There are other types of pharmacological interventions (e.g., aspirin, aminopyridine, and L-carnitine) being prescribed and tested for MS fatigue [52, 53]. Once the applicable trials that are currently underway are complete and the results become available, future reviews should be updated with a wider range of relevant terms to include those pharmacological interventions to present a more complete picture.

Unknown causes and mechanisms of MS fatigue, diverse consequences of MS fatigue, and the lack of precise methods of measuring the impact of MS fatigue all lead to our current challenge in developing, testing, and prescribing an effective intervention for people with MS who are experiencing disabling fatigue [48, 54]. Characteristics of MS fatigue vary. It is valuable for people with MS who are experiencing disabling fatigue to learn how to monitor the severity and/or the impact of fatigue and to select appropriate strategies to perform activities that are important to them. Exercise and pharmacological interventions are traditionally instructional (e.g., healthcare professionals instructing clients how much to exercise or what pill to take), and those included in this review are no exception. In comparison, the educational interventions in this review included self-management components (e.g., clients selecting strategies to manage fatigue based on their needs, environment, or preferences). Self-management approaches have been identified as highly effective in other chronic disease populations [55–57], which is consistent with the findings of this review.

Some studies suggest that heat and humidity, lack of sleep, or disturbed sleep aggravate MS fatigue [58–60]. Cooling therapies for managing heat intolerance, treatment for sleep problems (e.g., apnea), or educational programs for improving sleep hygiene and quality may also have potential for addressing MS fatigue. Nevertheless, we did not find RCTs of any of these interventions that met the review criteria when the literature search was conducted. In the future, examining these interventions in an updated review would be warranted to capture new evidence as it becomes available.

Systematic reviews and meta-analyses require clear a priori research questions. They are commonly conducted among trials with the same study design (e.g., RCT), intervention (e.g., surgery), and outcome (e.g., mortality). The setting (i.e., selecting the uniform design, intervention, and outcome) reduces the potential bias and improves the precision of the analysis, so that researchers can draw a firm conclusion about interventions. The difficulty of conducting such reviews and analyses for rehabilitation research has been acknowledged (e.g., due to the lack of RCTs) [61]. Even when the evidence is available, these RCTs often include small sample sizes, various intervention contents, and different outcomes. These qualities make it difficult for researchers to draw a well-supported conclusion about interventions. Some of the trials, particularly in the exercise interventions, had small sample sizes. Intervention contents and outcomes varied within each type of intervention (i.e., exercise, educational, and pharmacological). Although these characteristics among the included trials may be considered as other limitations of the review, the interventions do reflect current rehabilitation practice and therefore translate well from a clinical perspective.

MS care often looks to pharmacological agents for managing symptoms. These pharmacological agents, including fatigue medication, are important but often do not enable people with MS to cope with their existing disabilities. Fatigue is one of the most common and devastating MS symptoms affecting various components of one's health and wellbeing. People with MS experiencing fatigue and their healthcare providers should consider a full spectrum of effective fatigue management interventions, from exercise to educational strategies in conjunction with medication, to successfully manage the challenge.

## Figures and Tables

**Table 1:** A summary of three types of intervention trials.

	Exercise intervention trials [25–34]	Educational intervention trials [35–42]	Pharmacological intervention trials (Amantadine or Modafinil) [43–49]
*N* trials included in the review	10	8	7
*N* samples included in the analyses	233	662	604*
ES (range)	−0.24 to 2.05	−0.16 to 1.11	−0.59 to 0.55
Pooled ES (random effects)	0.57	0.54	0.07
95% CI for the pooled ES	0.10 to 1.04	0.30 to 0.77	−0.22 to 0.37
*P* value	0.02	<0.001	0.63
Heterogeneity	Yes (*Q* = 26.39, *P* = 0.003; *I* ^2^ = 65%)	Yes (*Q* = 14.14, *P* = 0.04; *I* ^2^ = 50%)	Yes (*Q* = 18.66, *P* = 0.004; *I* ^2^ = 67%)
Publication bias	No (Egger bias = 5.36, *P* = 0.13; Begg-Mazumdar = 0.29, *P* = 0.29)	No (Egger bias = 1.14, *P* = 0.54; Begg-Mazumdar = 0.21, *P* = 0.55)	No (Egger bias = −1.54, *P* = 0.57; Begg-Mazumdar = −0.24, *P* = 0.38)
*N* trials screened for the presence of fatigue as an eligibility criterion (%)	1 (10%)	6 (75%)	7 (100%)
Most common outcome used in the trials (*n*, %)	FSS (6, 60%)	MFIS (4, 50%)	FSS (5, 71%)
*N* of effective trials (%)	3 (30%)	6 (75%)	1 (14%)

*Include one trial reporting the outcome in a total cross-over sample combined together.

**Table 2:** Corresponding table for forest plots.

1st author	Experimental intervention	Comparison intervention	Outcome measure	ES	95% CI
Exercise intervention trials					
Kargarfard [25]	Aquatic exercise	No intervention	MFIS	2.05	1.00–3.11
Hebert [26]	Vestibular rehabilitation	Endurance and stretch exercise	MFIS	1.83	0.90–2.77
Velikonja [27]	Climbing	Yoga	MFIS	0.21	−0.69–1.11
Dalgas [28]	Progressive resistance training	No intervention	FSS	0.81	0.08–1.15
Mostert [29]	Bicycle aerobic exercise	No intervention	FSS	0.34	−0.43–1.11
van den Berg [30]	Treadmill Walking	No intervention	FSS	0.01	−0.96–0.99
Klefbeck [31]	Inspiratory muscle exercise	Usual care	FSS	1.01	−0.06–2.09
Cakt [32]	Cycling progressive resistance training	Home exercise for lower limb muscle strength and balance	FSS	0.20	−0.60–1.02
Hayes [33]	High intensity resistance training plus standard exercise	Standard exercise	FSS	−0.24	−1.15–0.64
Oken [34]	Aerobic exercise	Yoga	MFI	−0.17	−0.82–0.48
**Pooled ES**	**Random effects**			**0.57**	**0.10–1.04 (*P* = 0.02)**

Educational intervention trials					
Finlayson [35]	Fatigue management (teleconference)	Waitlisted	FIS	0.53	0.19–0.86
Mathiowetz [36]	Energy conservation course (in-person)	Waitlisted	FIS	0.42	0.08–0.76
Hugos [37]	Take control	Waitlisted	MFIS	0.43	−0.29–1.57
Grossman [38]	Mindfulness intervention	Usual care	MFIS	0.42	0.09–0.74
Kos [39]	Multidisciplinary Fatigue management	Placebo (nonfatigue focused program)	MFIS	−0.16	−0.72–0.38
Moss-Morris [40]	Cognitive Behavioral Therapy (CBT)	Standard care	MFIS	1.11	0.43–1.78
van Kessel [41]	CBT	Relaxation	FS	0.99	0.50–1.48
Mohr [42]	CBT	Group Psychotherapy	FAI	0.80	0.19–1.42
**Pooled ES**	**Random effects**			**0.54**	**0.30–0.77 (*P* < 0**.**001)**

Pharmacological intervention trials					
Shaygannejad [43]	Amantadine	Aspirin	FSS	−0.21	−0.76–0.32
Tomassini [44]	Amantadine	ALCAR	FSS	−0.59	−1.26–0.06
Ashtari [45]	Amantadine	Placebo	FSS	0.55	−0.06–1.16
Krupp^†^ [46]	Amantadine	Placebo	FSS	0.24	−0.23–0.73
The Canadian MS Research Group [47]	Amantadine	Placebo	VAS (0–50 mm)	0.21	−0.08–0.51
Möller [48]	Modafinil	Placebo	FSS	0.50	0.13–0.86
Stankoff^†^ [49]	Modafinil	Placebo	MFIS	−0.33	−0.70–0.02
**Pooled ES **	**Random effects**			**0.07**	−**0.22–0.37 (*P* = 0.63)**

^†^ES was estimated using the published graphical data presented in the article; CBT: cognitive behavioral therapy; MFIS: Modified Fatigue Impact Scale; FSS: Fatigue Severity Scale; MFI: Multidimensional Fatigue Inventory; FIS: Fatigue Impact Scale; FS: Fatigue Scale; FAI: Fatigue Assessment Instrument.

**Table 3:** Table of active interventions and their participants of the trials included in the review.

Study	Intervention		Participants					
1st author	Detail		Number of participants per group	Mean age	% women	Disability (EDSS)	MS type (% RRMS)	Mean years since diagnosis
Exercise intervention trials								
Kargarfard [25]	Aquatic exercise	60 min, 3*x*/week, 10 weeks	10	34	100	3	100%	5
Hebert [26]	Vestibular rehabilitation program, plus a 5 min fatigue education	60 min, 2*x*/week, 6 weeks	12	47	75	Criterion: able to walk 100 m	92%	6.5
Velikonja [27]	Climbing	1*x*/week, 10 weeks	10	42^†^	Unclear	4^†^	Unclear	Unclear
	Yoga	1/week, 10 weeks	10	41^†^		4^†^		
Dalgas [28]	Progressive resistance training	2*x*/week, 12 weeks	15	48	67	3.7	100%	7
Mostert [29]	Aerobic exercise (bicycle)	30 min, 5*x*/week, 3-4 weeks.	13	45	77	4.6	31%	11
van den berg [30]	Treadmill walking	Up to 30 min, 3*x*/week, 4 weeks	8 (immediate) 8 (delayed)	30–65 (range)	88%	Criterion: able to walk 10 meter <60 second	Unclear	Unclear
Klefbeck [31]	Inspiratory muscle training	70 sessions (total), 2*x*/day, 3-4/week, 10 weeks	7	46^†^	86	7.5^†^	Unclear	12^†^
Cakt [32]	Cycling progressive resistance training	2*x*/week, 8 weeks	14	36	64	Criterion: EDSS ≤ 6	Unclear	8
	Home-based exercise	2*x*/week, 8 weeks	10	43	80		Unclear	6
Hayes [33]	Resistance training (electronic ergometer)	45–60 min, 3*x*/week, 12 weeks	9	48	55.5	5	Unclear	12.5
	Standard exercise (aerobic, stretch, strengthening, and balance)	3*x*/week, 12 weeks	10	50	60	5	Unclear	12
Oken [34]	Yoga	90 min, 1*x*/week, 24 weeks	22	50	91	3.2	Unclear	Unclear
	Aerobic exercise (bicycle)	1*x*/week, 24 weeks	15	49	87	3		

Educational intervention trials								
Finlayson [35]	Teleconference fatigue management program	70 min, 1*x*/week, 6 weeks	89 (immediate) 92 (delayed)	56^x^	79^x^	4 (PDDS)^x^	52%^x^	15^x^
Mathiowetz [36]	Energy conservation course	120 min, 1*x*/week, 6 weeks	78 (immediate) 91 (delayed)	48^x^	83^x^	Unclear	61.5%^x^	9.5^x^
Hugos [37]	Take control program	120 min, 1*x*/week, 6 weeks	15	55	87	5	Unclear	14
Grossman [38]	Mindfulness intervention	150 min, 1*x*/week, 8 weeks; One 420 min session; 40 min/day homework	76	46	78	3	79%	8
Kos [39]	Multidisciplinary fatigue management	120 min, 1*x*/week, 4 weeks	28	43	71	Criterion: able to walk 100 meter without aid	72%	6
Moss-Morris [40]	CBT	25–50 min, 1*x*/week, 8–10 weeks	23	40	70	38% Able to walk 500 m or more	43.5%	21
van Kessel [41]	CBT	~50 min, 1*x*/week, 8 weeks	35	43	80	3	66%	5.5
	Relaxation therapy	~50 min, 1*x*/week, 8 weeks	37	47	70	4	49%	7
Mohr [42]	CBT	50 min, 1*x*/week, 16 weeks	22	45^x^	72^x^	2.5 (AI)^x^	Unclear	8.5^x^
	Supportive expressive group psychotherapy	90 min, 1*x*/week, 16 weeks	22					

Pharmacological intervention trials								
Shaygannejad [43]	Amantadine		26	36	85	1.5	85	3
Tomassini [44]	Amantadine		18	43	67	3	61	10
Ashtari [45]	Amantadine		21	26	33	2	100	6
Krupp [46]	Amantadine		31	40	68	3	90	11
The Canadian MS Research Group^x^ [47]	Amantadine		86	40	59	4	48	8
Möller [48]	Modafinil		62	41	63	3.5	47	7
Stankoff [49]	Modafinil		59	44	61	3.5	64	Unclear

^x^Data based on the entire efficacy study sample; ^†^Median; AI: Ambulation Index (mild to moderate gait impairment).

**Table 4:** Table of risk of bias assessment.

1st author	Selection bias	Allocation concealment	Blinding of participants and personnel	Blinding of outcome assessment	Incomplete outcome	Selective reporting
Exercise intervention trials						
Kargarfard [25]	U	L	H	H	L	L
Hebert [26]	U	U	H	H	L	L
Velikonja [27]	U	U	H	H	L	L
Dalgas [28]	U	U	H	H	L	L
Mostert [29]	U	U	H	H	L	L
van den Berg [30]	L	L	H	H	L	L
Klefbeck [31]	U	U	H	H	L	L
Cakt [32]	L	U	H	H	H	L
Hayes [33]	U	U	H	H	L	L
Oken [34]	L	L	H	H	L	L

Educational intervention trials						
Finlayson [35]	L	L	H	H	L	L
Mathiowetz [36]	L	U	H	H	L	L
Hugos [37]	L	L	H	H	L	L
Grossman [38]	L	L	H	H	L	L
Kos [39]	L	L	H	H	L	L
Moss-Morris [40]	L	L	H	H	L	L
van Kessel [41]	L	L	H	H	L	L
Mohr [42]	U	U	H	H	L	L

Pharmacological intervention trials						
Shaygannejad [43]	L	L	L	H	L	L
Tomassini [44]	U	U	L	H	H	L
Ashtari [45]	U	L	L	H	U	L
Krupp [46]	U	U	L	H	L	L
The Canadian MS Research Group [47]	L	U	L	H	L	L
Möller [48]	U	U	L	H	L	L
Stankoff [49]	U	L	L	H	L	L

H: high risk, L: low risk, U: unknown.

## Appendix

For more details see Tables 3 and 4.
